# Preclinical models for bladder cancer therapy research

**DOI:** 10.1097/MOU.0000000000001182

**Published:** 2024-04-16

**Authors:** Iris Ertl, Shahrokh F. Shariat, Walter Berger, Bernard Englinger

**Affiliations:** a Department of Urology, Comprehensive Cancer Center, Medical University of Vienna, Vienna, Austria; b Department of Urology, Weill Cornell Medical College, New York, New York; c Department of Urology, University of Texas Southwestern, Dallas, Texas, USA; d Department of Urology, Second Faculty of Medicine, Charles University, Prag, Czech Republic; e Karl Landsteiner Institute of Urology and Andrology, Vienna, Austria; f Research Center for Evidence Medicine, Urology Department Tabriz University of Medical Sciences, Tabriz, Iran; g Division of Urology, Department of Special Surgery, Jordan University Hospital, The University of Jordan, Amman, Jordan; h Center for Cancer Research, Medical University of Vienna, Vienna, Austria

**Keywords:** 3-dimensional bladder cancer cultures, patient-derived organoids, patient-derived xenografts, preclinical bladder cancer models, syngenic animal models

## Abstract

**Purpose of review:**

Bladder cancer (BC) is a highly heterogenous disease comprising tumours of various molecular subtypes and histologic variants. This heterogeneity represents a major challenge for the development of novel therapeutics. Preclinical models that closely mimic *in vivo* tumours and reflect their diverse biology are indispensable for the identification of therapies with specific activity in various BC subtypes. In this review, we summarize efforts and progress made in this context during the last 24 months.

**Recent findings:**

In recent years, one main focus was laid on the development of patient-derived BC models. Patient-derived organoids (PDOs) and patient-derived xenografts (PDXs) were demonstrated to widely recapitulate the molecular and histopathological characteristics, as well as the drug response profiles of the corresponding tumours of origin. These models, thus, represent promising tools for drug development and personalized medicine. Besides PDXs, syngenic *in vivo* models are of growing importance. Since these models are generated using immunocompetent hosts, they can, amongst others, be used to develop novel immunotherapeutics and to evaluate the impact of the immune system on drug response and resistance.

**Summary:**

In the past two years, various *in vivo* and *in vitro* models closely recapitulating the biology and heterogeneity of human bladder tumours were developed.

## INTRODUCTION

With an estimated 550.000 new cases and 200.000 deaths annually, bladder cancer (BC) ranks amongst the most common malignancies [1]. Despite recent breakthroughs, such as the approval of the pan-fibroblast growth factor receptor (FGFR) inhibitor erdafitinib and several immune checkpoint inhibitors (e.g. pembrolizumab, nivolumab, avelumab), as well as the development of antibody-drug conjugates (e.g. enfortumab vedotin), patients suffering from advanced and metastatic BC still have a dire prognosis [2–4].

A major hindrance for the development of novel therapies is the high heterogeneity of bladder tumours. While approx. 75% represent pure urothelial carcinoma (UC), the remaining 25% comprise various histologic variants such as squamous cell carcinoma (SCC), adenocarcinoma, or sarcomatoid carcinoma (SaC) [5]. Moreover, numerous studies showed that BC can be subdivided into various molecular subtypes with different sensitivities to currently available therapies [6,7]. 

**Box 1:**
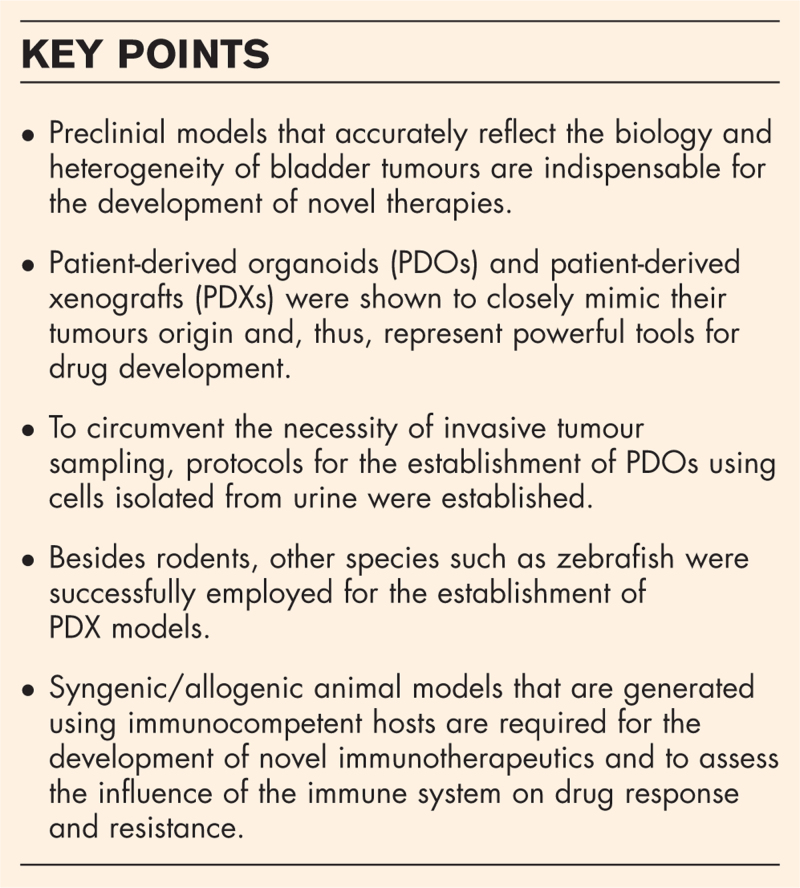
no caption available

**FIGURE 1:**
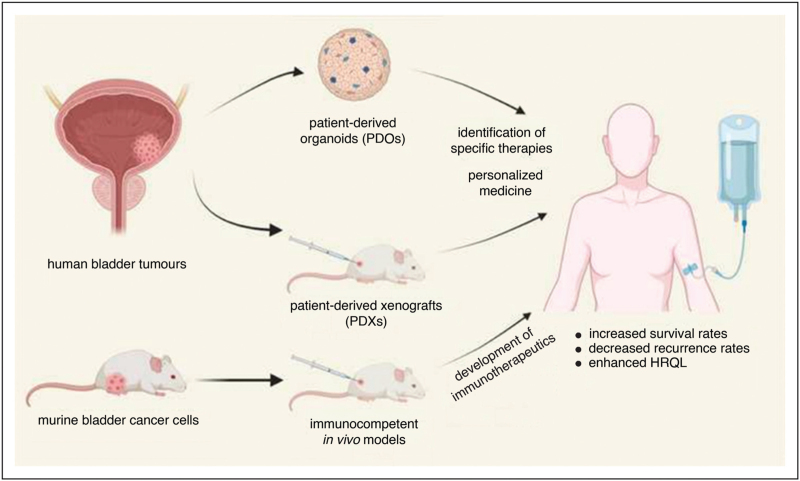
Overview of preclinical models for bladder cancer therapy research. Patient-derived *in vitro* and *in vivo* models recapitulate the molecular and histopathological characteristics, as well as the drug response profiles of the corresponding tumours of origin. Therefore, these models represent promising tools for drug development and personalized medicine. Syngenic/allogenic *in vivo* models that are generated using immunocompetent hosts are valuable tools for the development of novel immunotherapeutics. Thus, recently developed preclinical models have the potential to enable the development of novel BC treatments resulting in increased survival rates, decreased recurrence rates and enhanced health related quality of life (HRQL).

For the establishment of novel therapy schemes with specific activity in various BC subtypes, preclinical models that closely mimic *in vivo* tumours are indispensable. In this review, we summarize efforts and progress made in this context during the last 24 months. Given the literature published during this time period, we will mainly focus on 3-dimensional (3D) *in vitro* cultures, as well as on patient-derived xenograft and syngenic *in vivo* models (Fig. 1).

## *IN VITRO* MODELS

For several decades, *in vitro* studies for drug development were mainly conducted using 2D cultures of well established, commercially available BC cell lines. Even though these models represent powerful tools, they have considerable limitations affecting their clinical representativeness. On the one hand, classical BC cell lines have been cultured for many years, causing an accumulation of genetic and epigenetic alterations that arise with every passage [8,9]. One the other hand, traditional 2D models cannot recapitulate interactions between different cell types or cancer cells and the tumour microenvironment, that significantly influence drug response [10].

In the last 2 years, most research groups focused on the establishment of ‘organoids’ that are defined as 3D structures consisting of multiple types of cells [11]. Berndt-Paetz *et al.* developed organoids containing either of the classical BC cell lines RT4, RT112, T24 or CAL-29, as well as primary human bladder fibroblasts and smooth muscle cells. The organoids closely mimicked an inverse bladder wall, with a core of supportive cells surrounded by urothelial cells. Amongst other findings, the research group demonstrated significant differences in drug–response between organoids and conventional 2D cultures [12^▪^]. The superiority of 3-dimensional BC models in the context of drug development was also demonstrated by Wei *et al.*, who compared the sensitivities of BC cell lines and of patient-derived organoids (PDOs) to cisplatin, the Bcl-2 inhibitor venetoclax and the MCL1 inhibitor S63845 [13^▪^]. In an attempt to establish tools for a personalized treatment approach, Minoli *et al.* established an extensive collection of PDOs deriving from tumours of various disease stages and grades. The PDOs were found to accurately represent the molecular and histopathological characteristics and multiclonal landscapes of their tumours of origin. Testing the sensitivities of the PDOs to standard of care therapies, treatment outcomes observed in BC patient cohorts were accurately recapitulated. PDOs were, thus, shown to be a promising tool for the stratification of patients according to drug response sensitivity profiles [14^▪▪^]. Based on these findings, a clinical phase II trial with the aim to identify the most effective instillation therapy for individual NMIBC patients is presently being conducted [15^▪▪^]. Especially in the case of rare tumour entities, such as SaC, the lack of preclinical models impedes the development of specific drugs. To address this issue, Garioni *et al.* established a long-term organoid-like SaC model, as well as organoids representing conventional UC. A high-throughput drug screen including 1567 chemical compounds led to the identification of drug candidates with specific activity against either UC or SaC, and against both disease entities [16^▪^].

Organoid models also represent promising tools for the development of novel immunotherapeutics. Jiang *et al.* generated chimeric antigen receptor (CAR)-T cells targeting the transmembrane protein B7H3, which is highly expressed in most cancer types. Co-cultured PDOs underwent CAR-induced lysis, thus confirming specific antigen recognition and immune activation [17^▪^].

In order to establish a model system to study the role of immune cells in the response to anticancer compounds, Zhao *et al.* developed T-cell-retaining PDOs and investigated the effects of combination therapy with platinum (Pt) drugs and immune checkpoint inhibitors (ICIs). Several recently developed Pt^IV^ compounds were found to enhance the therapeutic efficiency of ICIs. Combinations of the Pt^IV^ compound Pt-19 and PD-1 inhibitors were further demonstrated to mediate the release of cytokines, the activation of immune-related pathways and the enhancement of T-cell receptor (TCR) clonal expansion [18^▪^].

Although PDOs generated from tumour tissue are powerful models for many aspects of BC research, they do not represent appropriate tools for longitudinal response monitoring and therapy adaption, since their establishment requires invasive tumour sampling. To circumvent this issue, Viergeber *et al.* developed a protocol for the establishment of BC organoids using cells isolated from urine of NMIBC and MIBC patients (’urinoids’). Immunohistochemistry confirmed identical histological features of urinoids, matching PDOs and corresponding tumours of origin. Analyzing samples of a single patient by Whole Genome Sequencing (WGS), it was demonstrated that the mutational profile of both urinoids and organoids were highly similar to the original tumour. Moreover, urinoids and organoids exhibited comparable sensitivities to various chemotherapeutics [19^▪▪^]. A similar protocol for the establishment of urine-derived BC organoids was published by Walz *et al.* The formation and primary expansion of urinoids was successful in 83% of cases, whereby the success rate was independent from factors such as sex and age of the patient, disease status and prior cancer-specific treatment [20^▪^]. Efforts were also made to develop novel methods for the accurate analysis of 3-dimensional BC models. Becker *et al.* established a label-free and noninvasive imaging procedure to monitor drug response combining Raman microspectroscopy (RMS) and fluorescence lifetime imaging microscopy (FLIM) [21]. Zhang *et al.* developed a deep learning model for the evaluation of the growth status of organoids called AU2Net (Attention and Cross U2Net) [22]. This algorithm is able to provide accurate segmentation results from organoid images, thus improving the analyses of drug screens using 3D cultures.

## *IN VIVO* MODELS

According to the location of the tumour, *in vivo* models are classified as heterotopic and orthotopic [23,24]. Orthotopic models can be generated by intravesical instillation or intramural injection of cancer cells, resulting in tumours located in the bladder. In heterotopic models, in contrast, tumour formation is achieved by subcutaneous injection of tumour cells [23]. Beyond that, *in vivo* models can further be categorized depending on the origin of the implanted cancer cells. While xenograft models are established by the implantation of human cancer cells into immunocompromised hosts, tumour formation in allogenic or syngenic models is induced by inoculation of tumour cells of the same species/strain in immunocompetent animals [24]. Besides the implantation of cancer cells, orthotopic tumours can also be induced by chemical carcinogenesis and genetic engineering [23].

### Transplantable tumour models

#### Xenograft models

Traditionally, xenograft animals used for drug development were established by implantation of classical BC cell lines. However, the clinical representativeness of cell line-based xenograft models is, amongst others, limited because they lack the heterogeneity of *in situ* patient tumours. In addition, cell lines lack their original stroma representing an important factor for tumour growth and drug response. This issue can be circumvented by usage of patient-derived xenograft (PDX) models that are generated by direct engraftment of tumour fragments into immunocompromised animals [24].

With the aim to facilitate the identification of novel therapies for muscle-invasive BC (MIBC) and upper tract urothelial carcinoma (UTUC) Lang *et al.* established an extensive panel of PDXs. Besides UTUC and conventional UC, the PDX collection also included various BC variants, such as SCC and SaC. Comparing patient tumours and PDXs, the researchers found a high resemblance concerning histological and genomic features, but alterations of the transcriptomic profiles that resulted – in some cases – in changes of intrinsic subtypes. However, since actionable mutations were retained, the PDX models widely recapitulated drug response observed in the clinics [25^▪▪^]. Steele *et al.* used PDXs to assess mechanisms of acquired cisplatin resistance. Thereby, the researchers found that treatment with the monoclonal ErbB3 antibody seribantumab resulted in significantly reduced tumour growth in both cisplatin resistant and sensitive models [26]. Laranjeira *et al.* employed PDXs to evaluate the effects of DNA methyltransferase inhibitors (DNMTi) on BC. Amongst others, it was shown that treatment with decitabine and 5-aza-4′-thio-2′-deoxycytidine (aza-T-dCyd) resulted in delayed tumour growth, inhibition of DNMT1 expression and upregulation of p21 [27].

Even though mice are the most common animal models in BC research, also other species can be used as hosts for human xenografts. Using fresh tumour samples, Kowald *et al.* created zebrafish tumour xenograft (ZTX) models for the prediction of BCG response. ZTX models were successfully established in 100% of cases (*n* = 6) and provided a correct prediction of treatment outcome for all patients that terminated the treatment (*n* = 4) [28^▪▪^]. Villanueva *et al.* aimed to optimize chicken embryo chorioallantoic membrane (CAM) PDX models for MIBC. Using cryopreserved or fresh tumour fragments, take rates of 32.1% and 69.3%, respectively, were achieved. By histological analysis, it was shown that undisrupted tissue fragments formed lesions highly resembling the tumours of origin. Treatment of CAM-PDX with cisplatin and gemcitabine confirmed that the models mirrored clinical therapy resistance [29^▪^].

#### Syngenic *in vivo* models

Xenograft models represent valuable tools for drug development. However, since they are generated using immunocompromised hosts, they cannot be used for the development of immunotherapies [24]. Given the increasing significance of immunotherapeutics for the treatment of BC, numerous studies using syngenic *in vivo* models were recently conducted.

Using the murine cell line MB49 and female C57BL/6 wild-type mice, Domingos-Pereira *et al.* generated an orthotopic model for non-muscle-invasive BC. Amongst others, tumour infiltration by myeloid derived suppressor cells (MDSC) and tumour associated macrophages (TAM) was studied and the expression of various chemokines and PD-1/PD-L1 was analysed. Thereby, it was shown that the established model represents a suitable tool for the development of ICIs and therapies targeting chemoattractants [30^▪^]. In order to study acquired resistance to ICIs, Denis *et al.* developed BC models by subcutaneous injection of MB49 and MBT-2 cells into C57BL6 or C3H/HeNRj mice, respectively. Resistance against anti–PD-1 and/or PD-L1 therapy was achieved by serial implantations and exposure to the respective antibodies. Amongst other findings, the gene *Serpinf1* was identified as a relevant factor in the resistance to anti-PD-1 antibodies [31^▪^]. To study mechanisms contributing to an immunosuppressive tumour microenvironment, Dominguez-Gutierrez *et al.* used organotypic tumour tissue slice cultures that were obtained from orthotopic BC models generated by subcutaneous injection of MBT-2 cells in C3/He mice. The approach revealed that hyaluronan (HA) produced in cell clusters in the stroma and tumour-draining lymph nodes supported the development of immunosuppressive PD-L1^+^ macrophages, thereby mediating immune escape and resistance to immunotherapy [32^▪^]. Xu *et al.* developed murine triple knockout (TKO: Trp53, Pten, Rb1) organoids that were injected subcutaneously, intravesically, or orthotopically into C57BL/6J mice. TKO tumours exhibited basal-like features and expressed, in contrast to lesions generated by implantation of MB49 cells, urothelial lineage markers. Treatment of subcutaneous TKO tumours with anti-PD-1 antibodies resulted in a mixed pattern of response reflecting immunotherapy responses observed in the clinics [33^▪^]. Tsuji *et al.* generated orthotopic BC models using the cell lines MBT-2 and MB49 and female C3H/HeJ or C57BL/6J mice to assess the effects of the novel bacterial minicell-based integrin-targeted oncolytic agent VAX014. In both models, intravesical administration resulted in significantly prolonged survival. Assessing combined treatment of VAX014 and systemic anti–PD-L1 therapy, synergies of the two agents were observed [34^▪^]. To study the effects of intratumoral xenogeneic urothelial cell (XUC) immunotherapy, Huang *et al.* generated syngenic mouse models by subcutaneous implantation of MBT-2 and MB49 cells. In both models, intratumoural injection of porcine urothelial cells resulted in the suppression of tumour growth that was further enhanced by chemotherapy (i.e., gemcitabine and cisplatin) [35^▪^].

To study the effects of intravesical adoptive cell therapy (ACT) with tumour infiltrating lymphocytes (TIL), Bazargan *et al.* generated an orthotopic model using C57BL/6 mice and ovalbumin (OVA) expressing MB49 cells (MB49OVA). Profiling the immune microenvironment, it was confirmed that the model recapitulated key characteristics of human tumours. Intravesical administration of gemcitabine was demonstrated to have lymphodepleting effects on the bladder microenvironment, thereby reducing immunosuppressive populations, such as myeloid derived suppressor cells (MDSCs). Pretreatment with gemcitabine was shown to result in enhanced antitumor effects of ACT [36^▪^].

To assess the effects of anti-CD40 immunotherapy on BC, Wong *et al.* generated orthotopic BC models using C57BL/6J mice and the cell lines MB49 and UPPL1541, of which the latter recapitulates the luminal subtype of high-grade UC. In the MB49 model, it was shown that intravesical treatment with anti-CD40 agonist antibodies caused reduced tumour burden, whereby the therapeutic effect was reduced by blockage of IL-15. In both the MB49 and UPPL1541 models, anti-CD40 agonist antibodies caused upregulation of IL-15Rα on dendritic cells (DCs) and other myeloid populations. Moreover, using CD40- and FcγR-humanized C57BL/6J mice harbouring MB49 or UPPL1541 tumours, it was shown that the human anti-CD40 agonist antibody 2141-V11 caused reduced tumour size and that this effect was enhanced by cotreatment with IL-15 [37^▪^].

### Carcinogen-induced *in vivo* models

A major factor affecting the pathogenesis of BC is chemical carcinogenesis, for instance due to smoking or occupational exposure [38]. Consequently, it is also possible to generate orthotopic BC models using carcinogenic agents [24]. Shah *et al.*, for instance, induced invasive tumours in male and female C57BL/6 mice by continuous exposure to N-butyl-N-(4-hydroxybutyl)-nitrosamine (BBN). Subsequently, tumours were passaged by subcutaneous implantation into sex matched C57BL/6 hosts. Histopathologic and molecular characterization showed that the eight established tumour lines represented basal/squamous (BaSq), stromal-rich (SR) and neuroendocrine (NE)-like molecular subtypes. As reported for human tumours, cisplatin exhibited antineoplastic effects in a SR tumour line, while a BaSq line was found to be resistant [39^▪^]. To assess the effects of the histone deacetylase (HDAC) inhibitor chidamide on BC, Wang *et al.* generated orthotopic models by instillation of N-methyl-N-nitrosourea (MNU) in the bladders of female Sprague–Dawley rats. Intravesical application of mitomycin C and chidamide alone or in combination revealed synergistic effects of the two drugs [40^▪^]. To assess the effects of inhibitors of the histone methyltransferase EZH2, Piunti *et al.* generated orthotopic tumour models by exposure of C57BL/6 mice to BBN. Application of the EZH2 catalytic inhibitor EPZ0011989 by oral gavage caused a reduction of tumour progression and increase immune infiltration. Further, it was shown that the antineoplastic effects of EZH2 inhibition depended on an intact adaptive immune system [41^▪^].

## CONCLUSION

The landscape of BC research is marked by significant advances in recent years, yet clear challenges persist. Despite advancements like FGFR inhibitors, ICIs and ADCs, the prognosis for advanced and metastatic BC remains bleak, with high heterogeneity further complicating therapeutic development. Efforts have intensified to address this complexity, with a particular focus on refining preclinical models to better mimic patient tumour biology.

While the discussed models offer promising avenues for bladder cancer research, they are not without limitations. Preclinical models, such as organoids and PDXs, may still inadequately capture the complexity of human tumours or long-term treatment effects, potentially leading to discrepancies in drug responses. Moreover, these models often require invasive procedures for tumour sampling, limiting their applicability for longitudinal studies. Furthermore, *in vivo* models face challenges in recapitulating e.g., human immune responses, limiting their utility in immunotherapy development. Addressing these limitations through ongoing refinement of models is crucial to improving their predictive accuracy and clinical utility.

Overall, the convergence of advanced preclinical models and innovative therapeutic strategies holds great promise for overcoming the challenges posed by BC heterogeneity and resistance mechanisms. Leveraging 3D organoids, PDXs, and syngenic/allogenic models promises to enhance drug discovery, personalized treatment approaches, and immunotherapy development. Continued interdisciplinary efforts in this direction are crucial for translating preclinical findings into clinically effective treatments, ultimately improving outcomes for patients battling this highly prevalent disease.

## Acknowledgements

*None.*

### Financial support and sponsorship

*None.*

### Conflicts of interest

*There are no conflicts of interest.*

## REFERENCES AND RECOMMENDED READING

Papers of particular interest, published within the annual period of review, have been highlighted as:

▪ of special interest
▪▪ of outstanding interest
